# Oral health during pregnancy: an analysis of interprofessional guideline awareness and practice behaviors among prenatal and oral health providers

**DOI:** 10.1186/s12884-023-06032-3

**Published:** 2023-10-11

**Authors:** Cheryl A. Vamos, Morgan Richardson Cayama, Helen Mahony, Stacey B. Griner, Rocio B. Quinonez, Kim Boggess, Jason Beckstead, Ellen M. Daley

**Affiliations:** 1 College of Public Health, University of South Florida, 13201 Bruce B. Downs Blvd MDC 56, Tampa, FL 33612 USA; 2 College of Social Sciences and Public Policy, Florida State University, 113 Collegiate Loop, Tallahassee, FL 32304 USA; 3 School of Public Health, The University of North Texas Health Science Center at Fort Worth, 3500 Camp Bowie Blvd Fort Worth, Fort Worth, TX 76107 USA; 4 Division of Pediatric Dentistry and Public Health, Department of Pediatrics, Schools of Dentistry and Medicine, University of North Carolina at Chapel Hill, 385 S. Columbia St., Chapel Hill, NC 27599 USA; 5 Department of Obstetrics and Gynecology, School of Medicine, University of North Carolina at Chapel Hill, 3009 Old Clinic Building, Campus Box 7570, Chapel Hill, NC 27599 USA

**Keywords:** Pregnancy, Oral health, Implementation science, Clinical guidelines

## Abstract

**Background:**

Poor oral health during pregnancy has significant implications across the life course, including increased risk for adverse pregnancy, birth outcomes, and the development of early childhood caries. In efforts to improve perinatal oral health in the United States, a set of national interprofessional guidelines were developed that include recommended practice behaviors for both oral health providers and prenatal providers. The purpose of this study was to examine guideline awareness, familiarity, beliefs, and practice behaviors among both provider types.

**Methods:**

Prenatal providers and oral health providers in Florida were recruited via random and convenience sampling to complete an online survey guided by the Consolidated Framework for Implementation Research (CFIR) and the Cabana Framework. The present analysis focused on the Individuals Involved domain (CFIR), awareness and familiarity with the guidelines (Cabana Framework), confidence, and practice behaviors as recommended by prenatal oral health guidelines (assess, advise, refer, share/coordinate). Data were analyzed using chi-square tests, independent samples t-tests, Pearson correlation coefficients, and one-way analysis of variance (ANOVA) and analyses were conducted in SPSS.

**Results:**

Prenatal and oral health providers did not differ significantly in their awareness of the guidelines, but awareness was significantly associated with three of the four practice behaviors for prenatal providers. Familiarity with the guidelines was significantly higher among oral health providers and was associated with all four practice behaviors for both provider types. Five out of ten oral health belief items were significantly associated with practicing the guidelines among prenatal providers, but only two among oral health providers. Confidence in performing the practice behaviors was significantly associated with guideline implementation among both groups. Years in practice was significantly associated with performing practice behaviors for prenatal providers, but not for oral health providers.

**Conclusions:**

Our findings highlight the importance of professional organizations and the role of clinical guidelines on practice behaviors. Although provider education is a key implementation strategy, organizational and policy-level system changes could also be critical in supporting practice behaviors.

## Introduction

The recent Oral Health in America publication [1], updating the work from the landmark 2000 Surgeon General’s Report on Oral Health in America [2], reconfirms the crucial role of oral health in overall health and wellbeing. This report affirmed the public health and life course significance of oral health, which has been linked to increased risk of systemic diseases (e.g., cardiovascular diseases, diabetes) and burdens on physical, mental, social, and economic well-being (e.g., stigma, missed school and work). Oral diseases are the most common chronic disease in the United States (U.S.) [3]. Missed opportunities for prevention costs the U.S. billions each year in treatment of dental conditions and other poor oral-systemic outcomes, burdening both patients and the healthcare system. These conditions are experienced disproportionately by socio-economically disadvantaged groups and underserved and underrepresented communities of color, driving persistent health disparities.

Within maternal and child health (MCH), poor oral health has implications across the life course [4]. Up to 75% of pregnant people develop gingivitis prior to birth, and poor maternal oral health has been linked with adverse pregnancy and birth outcomes (e.g., preterm birth and low birth weight) and early childhood caries [5, 6]. The nation’s Healthy People 2030 goals have identified 15 oral health-related objectives to combat poor oral health and increase dental care access [7]. Maternal and child oral health is also a priority in several states; for instance, in Florida, “improving dental care access for children and pregnant women” was identified as a priority in the state’s FY 2022 Title V application [8]. These objectives speak to the need for increased integration of oral health promotion during prenatal care, which has the potential to benefit the long-term health and well-being of women, children, and families.

Given the significance of oral health for MCH populations [4], professional associations have disseminated guidelines about prenatal oral health care [9, 10]. In 2012, a national consolidated set of interprofessional guidelines (co-sponsored by the American College of Obstetricians and Gynecologists and the American Dental Association) reiterated that all women should be provided oral health promotion and care during pregnancy [11]. Specifically, the guidance indicates that both provider types (prenatal and oral health providers) should (1) *assess*, (2) *advise*, (3) *refer*, and (4) *share/coordinate care*.

Nonetheless, data from the Pregnancy Risk Assessment Monitoring System (PRAMS) indicate that in 2020 only 40% of pregnant people in the U.S. had their teeth cleaned during pregnancy [12]. Further, those from socio-economically disadvantaged groups and communities of color have higher rates of untreated dental caries (i.e., cavities) [13]. There are also notable gaps in guideline awareness and implementation between prenatal and oral health providers. Research suggests that prenatal oral health education is taught widely in dental schools, but is lacking in obstetrics and gynecology residency programs [14]. Studies with prenatal providers demonstrate underutilization of oral health screenings, lack of referral to dental providers, and low awareness of importance of prenatal oral health [15–17]. Both provider types have been found to lack oral health knowledge and training, behavioral skills, time and motivation to successfully implement the prenatal oral heatlh guidelines into practice [17, 18].

Given significant gaps in translating guidelines into clinical practice, a comprehensive understanding of providers’ perspectives is essential to guide the development of future strategies that facilitate inter-professional guideline implementation across models of care. Subsequently, the aim of this study was to examine prenatal and oral health providers’ awareness, familiarity, beliefs, self-efficacy, and practice behaviors related to the interprofessional prenatal oral health guidelines.

## Methods

### Sampling and recruitment

The study sample included prenatal providers (CNM, MD, DO) and oral health providers (DMD, DDS) in the state of Florida. A combination of randomized and convenience sampling was used to recruit providers. Florida state licensure databases were used to obtain providers’ email addresses, which were then stratified by provider type, and block randomized. A modified Dillman tailored design approach [19] was then used to recruit potential participants, in which recruitment emails were sent by the Principal Investigator (PI) once every two weeks over the course of two months (four emails total). These emails included a description of the study and a link to the online survey. Additional recruitment efforts were conducted via state chapters of medical/dental professional organizations’ communication channels (e.g., professional membership newsletters, social media groups). Inclusion criteria were (1) at least 21 years of age; (2) hold one of the following credentials: MD, DO, CNM, DDS, or DMD; and (3) current licensed healthcare provider in the state of Florida. The survey took approximately ten minutes to complete, and participants were provided with a $10 e-gift card upon survey completion. This study was reviewed and approved by the University of South Florida’s Institutional Review Board.

### Survey Instrument

The study was guided by the Consolidated Framework for Implementation Research (CFIR) and the Cabana Framework. CFIR is meta-framework synthesizing 39 constructs from across established implementation theories and is often used to identify implementation barriers and facilitators [20]. The Cabana Framework, developed from a comprehensive review of barriers to clinical guideline adherence, was also used to better understand providers’ uptake of the guidelines [21]. Key barriers identified in the Cabana Framework include provider awareness and familiarity of the guidelines, agreement with the guidelines, self-efficacy, and external barriers [21]. The CFIR and Cabana Framework are complimentary, with the inclusion of intrapersonal determinants influencing providers’ practice behaviors (e.g., knowledge, self-efficacy); however, the Cabana Framework is specific to guideline adherence and distinguishes between guideline awareness and familiarity.

The process of survey development has been described elsewhere [22]. However, in brief, the survey was developed in consultation with a scientific advisory board (n = 3), comprised of researchers and clinicians with cross-cutting expertise in oral-systemic health, maternal and child oral healthcare, prenatal care, and implementation science. A practice advisory board (n = 8), consisting of practicing clinicians (two MDs, two DOs, two CNMs, two DDS, and two DMDs) engaged in a modified-Delphi technique process to identify high priority theory-driven constructs that would later inform survey items. The survey was pilot tested and refined using cognitive interviews with a convenience sample (n = 6) of prenatal (i.e., MD, CNM) and oral health providers (i.e., DMD) representative of the target population. The final survey examined beliefs, awareness and familiarity with the guidelines, self-efficacy (confidence) in guideline implementation, guideline implementation (performing recommended practice behaviors), and implementation barriers and facilitators. However, the analysis for this paper focuses on intrapersonal-level determinants of guideline implementation related to awareness, familiarity, beliefs, self-efficacy, and guideline implementation (practice behaviors) only. Demographic items include practice-focused questions, such as degree type, years in practice, practice setting, practice structure, and average number of patients seen weekly. Additional individual sociodemographic items, such as race, ethnicity, and gender were also included.

Awareness of the guidelines was measured by participants’ response to the item: *Are you aware of the existence of prenatal oral health guidelines?* Response options included yes, no, and not sure; for this analysis participants who answered either no or not sure were combined into a single category of no/not sure. Familiarity with the prenatal oral health guidelines was measured with the following item: *How familiar are you with the prenatal oral health guidelines*? Response options were on a five-point Likert scale ranging from (1) not at all familiar to (5) extremely familiar. Given the distribution of the responses, this item was treated as a continuous variable in this analysis and means and standard deviations are reported, with higher means indicating more familiarity with the guidelines.

Practice behaviors were measured by several items on the survey (Table 1). Composite variables were created by averaging the items used to measure *assess*, *advise*, and *share/coordinate*. [23] *Refer* was measured with only one item; thus, no composite variable was created for this outcome. Additionally, a meta-composite variable was created using an average of all practice behavior items to illustrate overall implementation of the guidelines. Response options for all items measuring practice behaviors were on a five-point Likert scale ranging from (1) never to (5) always.

**Table 1:** Participant and Practice Characteristics *Item was select all that apply

	Overall N = 275 N (%)	Prenatal N = 134 N (%)	Oral Health N = 141 N (%)	p-value
**Participant Characteristics**				
Primary Degree				
MD/DO	57 (20.7)	57 (42.5)		
CNM	77 (28.0)	77 (57.5)		
DMD	88 (32.0)		88 (62.4)	
DDS	53 (19.3)		53 (37.6)	
Gender				< 0.001
Male	109 (39.6)	31(23.1)	78 (55.3)	
Female	166 (60.4)	103 (76.9)	63 (44.7)	
Ethnicity				0.010
Hispanic or Latino	45 (16.4)	14 (10.4)	31 (22.0)	
Not Hispanic or Latino	230 (83.6)	120 (89.6)	110 (78.0)	
Race				0.007
White	220 (80.0)	112 (83.6)	108 (76.6)	
Black/African American	13 (4.7)	9 (6.7)	4 (2.8)	
Asian	16 (5.8)	2 (1.5)	14 (9.9)	
Other	26 (9.5)	11 (8.2)	15 (10.6)	
Age				< 0.001
18–29	18 (6.5)	5 (3.7)	13 (9.2)	
30–39	79 (28.7)	26 (19.4)	53 (37.6)	
40–49	61 (22.2)	31 (23.1)	30 (21.3)	
50–59	53 (19.3)	36 (26.9)	17 (12.1)	
60–69	52 (19.3)	32 (23.9)	21 (14.9)	
> 70	11 (4.0)	4 (3.0)	7 (5.0)	
**Practice Characteristics**				
Organization Type				
FQHC	49 (17.8)	34 (25.4)	15 (10.6)	0.001
Academic/Teaching	57 (20.7)	43 (32.1)	14 (9.9)	< 0.001
Larger Clinic Network	102 (37.1)	59 (44.0)	43 (30.5)	0.020
Insurance Accepted*				
Medicaid	144 (52.4)	111 (82.8)	33 (23.4)	< 0.001
Medicare	123 (44.7)	102 (76.1)	21 (14.9)	< 0.001
Tricare	160 (58.2)	103 (76.9)	57 (40.4)	< 0.001
Private	251 (91.3)	128 (95.5)	123 (87.2)	0.015
None/Sliding Scale	106 (38.5)	66 (49.3)	40 (28.4)	< 0.001
Self-Pay/OOP	256 (93.1)	125 (93.3)	131 (92.9)	0.902
Practice Structure				< 0.001
Solo	75 (27.3)	17 (12.7)	58 (41.1)	
Group	125 (45.5)	89 (66.4)	36 (25.5)	
Corporate	41 (14.9)	9 (6.7)	32 (22.7)	
Independent Contractor	15 (5.5)	5 (3.7)	10 (7.1)	
Hospitalist	8 (2.9)	8 (6.0)	0 (0.0)	
Other	11 (4.0)	6 (4.5)	5 (3.5)	
Practice Setting				< 0.001
Community Clinic	31 (11.3)	24 (17.9)	7 (5.0)	
Hospital	22 (8.0)	22 (15.4)	0 (0.0)	
Private Practice	202 (73.5)	81 (60.4)	121 (85.8)	
Health Department	7 (2.5)	2 (1.5)	5 (3.5)	
Other	13 (4.7)	5 (3.7)	8 (5.7)	
Average Total Patients/Week				0.353
1–20	19 (6.9)	9 (6.7)	10 (7.1)	
21–50	66 (24.0)	26 (19.4)	40 (28.4)	
51–70	67 (24.4)	34 (25.4)	33 (23.4)	
≥ 71	123 (44.7)	65 (48.5)	58 (41.1)	
Average Pregnant Patients/Week				< 0.001
1–10	150 (54.5)	18 (13.4)	132 (93.6)	
11–20	9 (3.3)	6 (4.5)	3 (2.1)	
21–50	66 (24.0)	65 (48.5)	1 (0.7)	
51–70	19 (6.9)	19 (14.2)	0 (0.0)	
≥ 71	23 (8.4)	23 (17.2)	0 (0.0)	
Do not see pregnant patients	8 (2.9)	3 (2.2)	5 (3.5)	

Belief questions focused on 10 items about oral health during pregnancy, including oral health and overall health, oral health care during pregnancy, and oral health hygiene behaviors. Participants were asked to indicate their agreement with each statement on a five-point Likert scale with response options ranging from (1) strongly disagree to (5) strongly agree. Given the distribution of the participant responses, these items were analyzed as continuous variables and mean agreement was reported for each statement, with higher means indicated more agreement with the statement. Three items were “false” and lower agreement with the statements indicated the participant believed the statement was incorrect; thus, these variables were reverse coded for analysis. Additionally, a composite variable of beliefs was created by taking an average of the 10 items.

Self-efficacy was operationalized as confidence and measured using 10 items pertaining to specific practice behaviors (e.g., confidence in checking pregnant patients’ mouths for problems) and overall behaviors of advising, referring, sharing, and coordinating care. Response options were on a five-point Likert scale from (1) not at all confident to (5) extremely confident. An overall composite variable of confidence in performing these behaviors was created using an average of all 10 items.

### Data Analysis

Listwise deletion was employed and only participants who had complete data for demographics and the variables relevant to the research questions were included in the analysis. Differences in demographic and practice characteristics by provider type were assessed using the Chi-square test. The independent samples t-test was used to examine if differences existed between provider types and familiarity with guidelines or years in practice. Pearson Correlation Coefficients were calculated to examine associations between familiarity of guidelines or beliefs with the practice behaviors (assess, advise, refer, share/coordinate). Likewise, associations between awareness of the guidelines and the practice behaviors were measured using ANOVA. A p-value of p < .05 was considered significant and all analyses were conducted in SPSS.

## Results

A total of 275 providers completed the survey, including 134 prenatal providers and 141 oral health providers. Provider and practice sociodemographic characteristics are presented in Table 1. Among prenatal providers, most were CNMs (57.5%), female (76.9%), and white (83.6%). Among oral health providers, the majority were DMDs (62.4%), male (55.3%), and white (78.0%). Prenatal providers in this sample had, on average, 17.96 (SD = 11.57) years in practice. Oral health providers had been practicing, on average, for 16.50 (SD = 13.22) years. The majority of prenatal providers worked in group (66.4%) and private practice (60.4%) settings, while most oral health providers worked in solo (41.1%) and private practice (85.8%) settings.

### Awareness

Fifty-seven percent and 65% of prenatal and oral health providers were aware of the guidelines, respectively, with no significant difference between these two groups (p = .147). Differences in guideline awareness were then assessed by provider type in relation to practice behaviors for both prenatal and oral health providers. In regard to practice behaviors, there was a statistically significant difference between prenatal providers who were aware versus those unaware of the guidelines as determined by one-way ANOVA.^a^ Prenatal providers who were aware of the guidelines had statistically significant higher means for the practice behaviors of Advise (F(2, 131) = 16.531, p < .001), Assess (F(2, 131) = 13.827, p < .001), and Refer (F(2, 131) = 7.698, p = .006). In contrast, no practice behaviors were significantly associated with awareness among oral health providers.

### Familiarity

Prenatal providers (M = 2.31, SD = 1.03) and oral health providers (M = 2.75, SD = 1.14) reported only being slightly to moderately familiar with the guidelines. Statistical differences between provider types regarding familiarity was assessed using a one-way ANOVA, which demonstrated that the mean familiarity for oral health providers was significantly higher than that of prenatal providers (F(1, 273) = 11.621, p = .001). Associations between familiarity with the guidelines and the four key practice behaviors were assessed using Pearson Correlation Coefficients. Familiarity with the guidelines was significantly associated with all practice behaviors for both provider types. Among prenatal providers, these correlations were as follows: Assess (r = .566, p < .001), Advise (r = .532, p < .001), Refer (r = .337, p < .001), and Share/Coordinate (r = .429, p < .001). Among oral health providers, these were: Assess (r = .199, p = .018), Advise (r = .273, p = .001), Refer (r = .305, p < .001), and Share/Coordinate (r = .301, p < .001).

### Beliefs

On average, both provider types reported that they strongly agreed with all belief items (Table 2). Prenatal providers reported most strongly agreeing with the statement, “Seeking oral health care is safe during pregnancy,” (M = 4.75, SD = 0.77) and reported the lowest agreement with the item, “Mothers can transmit the bacteria that causes cavities to their infants by kissing or sharing spoons,” (M = 3.33, SD = 1.15). Oral health providers reported the strongest agreement with the statement, “Oral health is important for the overall health of my pregnant patients,” (M = 4.84, SD = 0.64) and the lowest with “My professional organization has guidance on oral health promotion and care during pregnancy,” (M = 4.18, SD = 1.02).

**Table 2:** Means of belief and confidence items for each provider type

**Belief Items** ^**a**^	**Prenatal Provider** **M (SD)**	**Oral Health Provider** **M (SD)**
Oral health is important for the overall health of my pregnant patients	4.56 (0.96)	4.84 (0.64)
Seeking oral health care during pregnancy is recommended	4.65 (0.99)	4.71 (0.79)
Mothers can transmit the bacteria that causes cavities to their infants by kissing or sharing spoons	3.33 (1.15)	4.33 (1.04)
Physiological changes in the mouth can occur during pregnancy	4.58 (0.78)	4.74 (0.61)
Poor oral health during pregnancy is associated with adverse pregnancy and birth outcomes	4.46 (0.79)	4.19 (0.96)
A mother’s oral health status is associated with her children’s oral health status	4.04 (0.88)	4.26 (0.92)
My professional organization has guidance on oral health promotion and care during pregnancy	3.75 (1.13)	4.18 (1.02)
Oral health counseling during pregnancy may help to reduce the transmission of cavity-associated bacteria from mothers to infants	3.91 (0.85)	4.31 (0.84)
Seeking oral health care is safe during pregnancy	4.75 (0.77)	4.73 (0.71)
Mothers’ oral health hygiene behaviors are associated with their children’s oral health hygiene behaviors	4.52 (0.69)	4.60 (0.70)
**Confidence Items** ^**b**^	**Prenatal Provider** **M (SD)**	**Oral Health Provider M (SD)**
Taking an oral health history from pregnant patients	3.37 (1.08)	4.62 (0.71)
Checking pregnant patients’ mouths for problems	3.07 (1.18)	4.70 (0.64)
Documenting oral health assessment findings in pregnant patients’ medical records	3.20 (1.13)	4.66 (0.63)
Advising pregnant patients that oral healthcare is safe during pregnancy	4.40 (0.84)	4.59 (0.70)
Advising pregnant patients on good oral hygiene	4.31 (0.88)	4.79 (0.53)
Advising pregnant patients to eat healthy foods	4.63 (0.68)	4.50 (0.82)
Advising pregnant patients to seek oral healthcare	4.37 (0.91)	3.94 (1.15)
Referring pregnant patients to an oral health provider	3.91 (1.15)	3.48 (1.37)
Sharing information about pregnant patients with oral health providers/prenatal providers^c^	3.34 (1.23)	3.32 (1.39)
Coordinating care for pregnant patients with oral health providers/prenatal providers^c^	3.73 (1.01)	3.63 (1.26)

^a^ Response options range from (1) strongly disagree to (5) strongly agree

^b^ Response options range from (1) not at all confident to (5) extremely confident

^c^ Survey language varied depending on provider type

Among prenatal providers, five out of 10 belief items were associated with one or more practice behaviors (Table 3). Only one item was significantly associated with Assess and two items each with either Advise, Refer or Share/Coordinate. Among oral health providers, six belief items were associated with Assess, eight were associated with Advise, and one item each was associated with either Refer or Share/Coordinate. Positive correlation coefficients indicate that the higher agreement with the belief items, the more likely participants were to engage in the practice behaviors. Associations between all ten belief items and the composite practice behavior variable (including all practice behavior items) were also assessed for both provider types. Among prenatal providers, only two of the ten beliefs were significantly associated with the practice composite score. Among oral health providers, seven beliefs were significantly associated with the practice composite score.

**Table 3:** Significance of belief items with practice behavior for each provider typea

	Prenatal Provider r (p-value)	Oral Health Provider r (p-value)
Question 1. Oral health is important for the overall health of my pregnant patients.		
Assess	0.055 (0.524)	**0.304 (< 0.001)**
Advise	0.153 (0.078)	.**271 (0.001)**
Refer	0.034 (0.697)	0.098 (0.250)
Share/Coordinate	0.025 (0.772)	0.111 (0.191)
Practice Composite	0.093 (0.283)	**0.275 (< 0.001)**
Question 2. Seeking oral health care during pregnancy is recommended.		
Assess	− 0.090 (0.299)	0.103 (0.225)
Advise	0.012 (0.89)	0.048 (0.572)
Refer	− 0.137 (0.115)	− 0.042 (0.622)
Share/Coordinate	− .2**41(0.005)**	− 0.002 (0.985)
Practice Composite	− 0.112 (0.198)	0.040 (0.637)
Question 3. Mothers can transmit the bacteria that causes cavities to their infants by kissing or sharing spoons.		
Assess	0.072 (0.410)	0.146 (0.084)
Advise	0.029 (0.736)	0.151 (0.074)
Refer	0.106 (0.224)	0.118 (0.164)
Share/Coordinate	0.105 (0.226)	0.068 (0.421)
Practice Composite	0.081 (0.352)	0.164 (0.052)
Question 4. Physiological changes in the mouth can occur during pregnancy.		
Assess	0.056 (0.519)	**0.389 (< 0.001)**
Advise	0.061 (0.483)	**0.350 (< 0.001)**
Refer	0.100 (0.248)	0.011 (0.894)
Share/Coordinate	0.027 (0.757)	0.052 (0.543)
Practice Composite	0.066 (0.448)	.**289 (< 0.001)**
Question 5. Poor oral health during pregnancy is associated with adverse pregnancy and birth outcomes.		
Assess	0.068 (0.437)	**0.210 (0.013)**
Advise	0.107 (0.218)	**0.304 (< 0.001)**
Refer	**0.232 (0.007)**	**0.193 (0.022)**
Share/Coordinate	0.097 (0.267)	0.121 (0.153)
Practice Composite	0.123 (0.156)	**0.288 (< 0.001)**
Question 6. A mother’s oral health status is associated with her children’s oral health status.		
Assess	0.062 (0.476)	**0.211 (0.012)**
Advise	− 0.039 (0.657)	**0.253 (0.002)**
Refer	− 0.001 (0.989)	0.085 (0.315)
Share/Coordinate	− 0.006 (0.943)	0.064 (0.453)
Practice Composite	0.008 (0.926)	**0.218 (0.010)**
Question 7. My professional organization has guidance on oral health promotion and care during pregnancy.		
Assess	**0.230 (0.007)**	0.144 (0.089)
Advise	**0.291 (< 0.001)**	**0.293 (< 0.001)**
Refer	0.116 (0.183)	0.016 (0.848)
Share/Coordinate	0.111 (0.201)	0.107 (0.207)
Practice Composite	**0.251 (0.003)**	**0.216 (0.010)**
Question 8. Oral health counseling during pregnancy may help to reduce the transmission of cavity-associated bacteria from mothers to infants.		
Assess	0.163 (0.059)	**0.173 (0.040)**
Advise	**0.250 (0.004)**	**0.260 (0.002)**
Refer	**0.284 (< 0.001)**	0.142 (0.093)
Share/Coordinate	0.155 (0.074)	0.161 (0.056)
Practice Composite	**0.240 (0.005)**	**0.263 (0.002)**
Question 9. Seeking oral health care is safe during pregnancy.		
Assess	− 0.049 (0.577)	**0.364 (< 0.001)**
Advise	0.016 (0.851)	**0.295 (< 0.001)**
Refer	− 0.020 (0.816)	− 0.108 (0.202)
Share/Coordinate	**− 0.196 (0.023)**	**− 0.203 (0.016)**
Practice Composite	− 0.067 (0.440)	0.124 (0.142)
Question 10. Mothers’ oral health hygiene behaviors are associated with their children’s oral health hygiene behaviors.		
Assess	0.036 (0.679)	0.165 (0.051)
Advise	0.021 (0.809)	**0.234 (0.005)**
Refer	0.026 (0.769)	0.122 (0.149)
Share/Coordinate	− 0.063 (0.467)	0.088 (0.298)
Practice Composite	0.009 (0.914)	**0.215 (0.011)**

*Note*: Items in bold denote significance at p < .05

^a^ Response options range from (1) strongly disagree to (5) strongly agree

### Confidence

Prenatal providers reported the highest confidence with the following behaviors: advising pregnant patients to eat health foods (M = 4.63, SD = 0.68) and the lowest with checking pregnant patients’ mouths for problems (M = 3.07, SD = 1.18) (Table 2). Oral health providers reported the highest confidence with advising pregnant patients on good oral hygiene (M = 4.79, SD = 0.53) and the lowest with sharing information about pregnant patients with prenatal providers (M = 3.31, SD = 1.39). On average, prenatal providers reported being moderately to very confident in performing recommended practice behaviors (M = 3.83, SD = 0.72) while oral health providers reported being very confident (M = 4.22, SD = 0.69). Among both provider types, overall confidence in performing recommended practice behaviors was significantly associated with their frequency of performing each behavior and their overall implementation of the guidelines, denoted by the practice composite variable (Table 4).

**Table 4:** Significance of confidence with practice behaviors for each provider typea

Overall confidence in performing recommended practice behaviors		
	Prenatal Provider r (p-value)	Oral Health Provider r (p-value)
Assess	0.551 (< 0.001)	0.449 (< 0.001)
Advise	0.479 (< 0.001)	0.504 (< 0.001)
Refer	0.412 (< 0.001)	0.283 (< 0.001)
Share/Coordinate	0.454 (< 0.001)	0.363 (< 0.001)
Practice Composite	0.581 (< 0.001)	0.566 (< 0.001)

^a^ Response options from (1) not at all confident to (5) extremely confident

### Years in practice

For prenatal providers, years in practice was significantly associated with assess (r = .285, p < .001), advise (r = .273, p = .001), refer (r = .231, p = .007), and share/coordinate (r = .267, p = .002). Positive correlation coefficients indicate that more years of practice was associated with engaging in the practice behaviors. No significant associations were found for years in practice and awareness, familiarity, or beliefs.

Among oral health providers, years in practice was associated with the following two belief items: (1) Mothers can transmit the bacteria that causes cavities to their infants by kissing or sharing spoons (r = − .167, p = .048); and (2) Seeking oral health care is safe during pregnancy (r = .210, p = .013). A negative correlation coefficient indicates that the more years in practice that oral health providers have, the lower their agreement with this item. Years in practice was not significantly associated with awareness, familiarity, or practice behaviors.

## Discussion

This study aimed to examine prenatal and oral health providers’ awareness, familiarity, beliefs, self-efficacy and practice behaviors as identified in the interprofessional prenatal oral health guidelines (i.e., assess, advise, refer, share/coordinate). In this sample, only between 57 and 65% of providers were *aware* of the guidelines, with oral health providers reporting a statistically significant higher level of awareness. Among prenatal providers, guideline awareness was significantly associated with practice behaviors (assess, advise and refer), but these associations were not significant for oral health providers. Our finding of low guideline awareness is similar to prior research. In a study examining the awareness of state prenatal oral health promotion guidelines, 45% of OBGYN program directors and 65% of dental school deans reported guideline awareness [24]. In addition, a qualitative study of 22 prenatal and oral health providers found that the majority were unaware of the guidelines [18].

Although one could be aware of the existence of guidelines, being *familiar* with the specific evidence and recommendations is needed to support actual behavior change. In this study, both provider types were only slightly to moderately *familiar* with the guidelines, with oral health providers reporting a statistically significant higher mean familiarity compared to prenatal providers. Greater familiarity among oral health providers is perhaps unsurprising given that the guidelines focus on oral health behaviors, such as assessing patients’ mouths for problems and advising patients on oral health care which is a basic scope of practice for oral health providers. Nonetheless, guideline familiarity was significantly associated with all practice behaviors (assess, advise, refer, share/coordinate) among both provider types, demonstrating the importance of guideline familiarity in influencing clinical practice behaviors. Low familiarity with guidelines has been found in studies across a range of health care providers, in addition to low familiarity specific to the prenatal oral health guidelines among prenatal and oral health providers [18, 25–27]. For example, in one study, lack of guideline familiarity was a primary barrier to physician adherence [25], while in another study, physician familiarity with five common clinical guidelines was significantly associated with practice behavior change relating to two of the guidelines [27].

Both provider types in this present study reported relatively high agreement with all *belief* items demonstrating high knowledge of oral health during pregnancy. In addition, specific individual oral health belief statements and the composite belief score were significantly associated with various practice behaviors among both provider types. Similar to our study, other researchers have found mixed results regarding provider beliefs and prenatal oral health. For example, oral health providers more often discussed the belief that there is a connection between oral health and pregnancy outcomes compared to prenatal providers [18]. On the contrary, a study conducted among OBGYN’s in Ohio found that providers were more likely to have accurate beliefs regarding the safety of seeing a dentist during any trimester and whether there were associations between oral health and pregnancy compared to dentists [28]. However, among the 66% of OBGYN’s who reported a need for dental care during pregnancy, only 29% performed an oral exam, 20% used oral health screening questions, and 6% referred their patients to a dentist [28]. It is interesting to note that the belief statement in the current study, “*Mothers can transmit the bacteria that causes cavities to their infants by kissing or sharing spoons.*” was not associated with any practice behavior. This could be due to evolving science around the microbiome and epigenetics and the complicated mechanisms by which bacteria are transmitted from the primary caregiver to an infant [29].

Prenatal providers reported being only moderately *confident* with many recommended practice behaviors, particularly those associated with assessing pregnant patients’ oral health. As might be expected, oral health providers reported being very to extremely confident with these behaviors. Both provider types, however, reported being moderately to very confident in sharing information about and coordinating care for pregnant patients with the other provider type and providing referrals. Overall self-efficacy in performing the recommended practice behaviors was significantly associated with reported frequency of performing these behaviors for both provider types. Medical providers have previously reported lack of oral health training in their training programs and generally low levels of comfort with identifying signs of oral pathology and examining tooth decay [30]. A study exploring interprofessional collaboration between dental and primary care providers found that very few primary care providers reported receiving training in oral health, and that previous training was significantly associated with providers’ confidence in examining patients’ oral cavity [31]. Thus, given the strong and significant association between providers’ self-reported confidence and their frequency of performing these behaviors, both provider types may benefit from increased education and training to ensure oral health guideline implementation. Moreover, the number of years in practice was significantly positively associated with all recommended practice behaviors among prenatal providers but was not significant for oral health providers, suggesting that different provider education training strategies may need to be tailored for emerging providers (i.e., medical and dental programs) compared to those already in the workforce. Assessing current medical school curricula and residency programs and identifying gaps in didactic and skills training could increase practice behaviors [24]. Additionally, facilitating interprofessional training could potentially address the lack of practice behaviors [32–34]. Lastly, provider education is often utilized as a key implementation strategy [35], however, the evidence around which training techniques are most accepted, useful, and effective among healthcare providers in the field remains understudied.

This study focused on intrapersonal level determinants of practice behaviors, and thus future research should explore additional system-level determinants influencing guideline implementation. Theories and methods derived from the field of dissemination and implementation sciences can guide such efforts and assist with aligning implementation strategies to maximize facilitators and address barriers in practice [36, 37]. Dissemination and implementation theories and methods can also take into account the different organizational contexts unique to medical and dentistry models that may influence adoption, institutionalization, and sustainability of interventions designed to support guideline implementation. For instance, research could examine how organizational-level determinants, such as whether a practice has an institutional policy and procedure, training, resources and supports, and a champion for guideline implementation may facilitate prenatal oral health guideline practice behaviors. In addition, there is also a need for the development of interventions to incorporate a user-centered design approach [38], to assure that provider- and person-centered interventions are focused on the needs and preferences of end-users. Moreover, the belief regarding whether professional associations have guidelines for prenatal oral health was significantly associated with practice behaviors. This calls attention to the need to better understand effective dissemination efforts that serve as critical prerequisites before implementation strategies can be designed. Future research should highlight the important role of professional organizations in effectively reaching providers with current policies, guidelines, best practices, and other supports, particularly as they relate to inter-professional collaborations on patient care.

Findings from this study should be considered in light of its limitations. Given the mixture of random and convenience sampling techniques employed, the response rate is unknown and there could be response bias present where those providers who hold particular views or experiences may have elected to participate. In addition, only practicing prenatal providers (CNM, MD, DO) and oral health providers (DMD, DDS) in the state of Florida were eligible, and other key clinical team members (e.g., nurses and dental hygienists) who are often engaged in practice behaviors (e.g., patient education, assessment, and referrals) were not included. Therefore, future research should include other clinical team members from other regions of the U.S. to improve the generalizability of findings. Nonetheless, this study was comprised of several strengths, such as being driven by evidence (guidelines) and implementation theory (CFIR, Cabana Framework), supported by guidance by Scientific and Practice Advisory Boards, and can contribute to the field of dissemination and implementation sciences as it relates interprofessional practices.

In conclusion, findings from this study found significant gaps in prenatal and oral health providers’ awareness, familiarity, beliefs, and practice behaviors related to the interprofessional prenatal oral health guidelines. Although provider education is a key implementation strategy, organizational and policy-level system changes may also be critical to support practice behaviors during clinical encounters. Such efforts are essential in the ultimate goal of promoting oral-systemic health among MCH populations across the life course.

## Data Availability

The dataset from this study is available from the corresponding author upon reasonable request.
